# Fine-scale structural diversification among closely related *Legionella pneumophila* isolates from a bath-associated environmental reservoir

**DOI:** 10.1128/aem.01127-26

**Published:** 2026-08-28

**Authors:** Naoki Nakajima, Michio Jinnai, Aya Okamura, Yoshimi Date, Ichiro Furukawa

**Affiliations:** 1 Department of Microbiology, Kanagawa Prefectural Institute of Public Health 91350, Kanagawa, Japan; 2 Division of Biomedical Food Research, National Institute of Health Sciences 243142, Kawasaki, Japan; University of Minnesota Twin Cities, St. Paul, Minnesota, USA

**Keywords:** *Legionella pneumophila*, insertion sequence, IS256, whole-genome sequencing, comparative genomics, structural variation

## Abstract

*Legionella pneumophila* populations within environmental reservoirs can exhibit genomic diversity, complicating source attribution during outbreak investigations. Core genome single-nucleotide variant (cgSNV)-based approaches are widely used for identifying closely related lineages during molecular epidemiological investigations; however, they primarily focus on nucleotide-level variation and may overlook structural variation among closely related isolates. In this study, we investigated genomic diversity among clinical and environmental *L. pneumophila* isolates obtained during an epidemiological investigation of a legionellosis case. By analyzing multiple colonies isolated from individual samples, we integrated cgSNV analysis with comparative genome structural analysis to characterize insertion sequence (IS)-mediated structural variation within a highly related population. A total of 138 serogroup 1 isolates belonging to sequence type 138 were analyzed. cgSNV analysis revealed that clinical isolates formed a clonal lineage, whereas environmental isolates from the same bath system exhibited substantial diversity despite differing by no more than 20 cgSNVs. Complete genome analysis of 48 representative isolates demonstrated nearly identical genome synteny with limited large-scale rearrangements. In contrast, IS insertion patterns showed considerable variability, particularly for IS256, which exhibited extensive diversity in insertion sites across isolates. Differences in IS insertion patterns were observed even among isolates sharing identical cgSNV genotypes, indicating that IS-mediated structural variation can occur independently of core genome SNV variation. More than half of the coding sequence differences relative to the reference genome were associated with IS insertion sites, predominantly IS256-associated events. These findings suggest that closely related *L. pneumophila* populations within environmental reservoirs undergo fine-scale diversification associated with dynamic IS activity.

**IMPORTANCE:**

In this study, we analyzed a large collection of *Legionella pneumophila* isolates obtained from a patient and a bath-associated environmental source and identified extensive insertion sequence-associated structural variation even among isolates sharing highly similar core genome single-nucleotide variant genotypes. Despite this structural diversification, the closely related isolate population retained highly conserved genome organization and broadly similar insertion sequence distribution patterns, supporting the interpretation that it remained highly clonal rather than undergoing large-scale genomic divergence. These findings highlight the importance of analyzing multiple colonies from individual samples to better characterize within-source population diversity. They further indicate that structural genome variation, particularly mobile insertion sequences such as IS256, provides complementary resolution beyond conventional nucleotide-based approaches for resolving fine-scale relationships among closely related isolates. Our study provides new insight into the microevolution of *L. pneumophila* populations within environmental reservoirs relevant to public health investigations.

## INTRODUCTION

*Legionella pneumophila* is a ubiquitous environmental bacterium that inhabits natural and artificial aquatic systems, including cooling towers and plumbing systems (1, 2). Human infection typically occurs through inhalation of contaminated aerosols, with environmental water systems serving as the primary reservoirs of *L. pneumophila* (3, 4). In Japan, public bathing facilities have been identified as a major source of infection (5, 6), underscoring the importance of identifying environmental reservoirs and understanding the population structure of *L. pneumophila* within these systems for effective public health interventions.

Recent advances in whole-genome sequencing, particularly short-read sequencing-based approaches, have substantially improved the resolution of molecular epidemiological investigations of *L. pneumophila*. These studies have revealed considerable genetic diversity among environmental *L. pneumophila* populations and have demonstrated that multiple closely related genotypes can coexist within a single outbreak source or environmental reservoir (7, 8). In addition, *Legionella* species are known to possess highly plastic genomes shaped by frequent recombination, horizontal gene transfer, and mobile genetic elements, all of which contribute to their genomic evolution and adaptation (9–11).

Given these genomic characteristics, outbreak sources are not necessarily composed of a single clone, and core genome single-nucleotide variant (cgSNV)-based approaches have become increasingly important for estimating genetic relatedness among isolates and identifying infection-associated lineages during epidemiological investigations (12, 13). Because environmental populations of *L. pneumophila* are genetically diverse, analyses based on only a limited number of isolates may fail to identify environmental isolates that are nearly identical to the corresponding clinical isolates, underscoring the importance of analyzing multiple clinical and environmental isolates (14, 15). Nevertheless, in many previous studies, the number of colonies analyzed per sample has remained limited, potentially leading to underestimation of within-source genomic diversity. Moreover, although cgSNV-based approaches are highly effective for identifying closely related lineages, they primarily focus on nucleotide-level variation. Consequently, they may overlook structural variation, including differences in mobile genetic elements such as insertion sequences (ISs), among closely related isolates coexisting within the same environmental reservoir.

Advances in long-read sequencing technologies have enabled the generation of complete bacterial genome assemblies, overcoming limitations of short-read sequencing in repetitive genomic regions and allowing detailed characterization of structural variation, including inversions, insertions, duplications, and mobile genetic element-mediated rearrangements (16, 17). Among these mobile elements, ISs are increasingly recognized as major drivers of bacterial genome plasticity and evolution, contributing to structural variation, gene disruption, genome rearrangement, and adaptive diversification (18, 19). Complete genome-based structural analyses of *Legionella* isolates have demonstrated long-term persistence of highly related clones within environmental water systems over multiple years (20, 21). However, these approaches have not yet been extensively applied to characterize diversity among colonies isolated simultaneously from the same environmental sample. Consequently, despite the recognized genomic plasticity of *L. pneumophila*, little is known about how IS dynamics contribute to fine-scale genomic diversity among closely related isolates coexisting within infection-associated environmental reservoirs.

This study was based on an epidemiological investigation of a legionellosis case in Japan involving a single patient who had used a bath facility. During the investigation, *L. pneumophila* was isolated from the patient and environmental samples, including bathwater and bathtub swabs collected at the facility. Multiple colonies were isolated from each sample to comprehensively investigate within-source genomic diversity. By integrating cgSNV analysis with comparative genome structural analysis based on complete genome sequences, we aimed to characterize IS dynamics and structural variation within a highly related population. Specifically, we investigated whether IS-mediated structural variation contributes to within-source genomic diversity beyond core genome SNVs and provides additional resolution for characterizing genomic diversity among closely related isolates within an environmental reservoir. In addition, using a *Galleria mellonella* infection model for the assessment of *Legionella* virulence (22), we investigated whether IS-mediated structural variation was accompanied by detectable differences in virulence among representative isolates with different genomic structural features.

## RESULTS

### Isolation overview

One sample of patient sputum, one of bathwater from bathtub A, and one surface swab each from the edges of bathtubs A and B were collected. The patient had used the bathing facility approximately 9 days before sputum collection, whereas environmental samples were collected approximately 13 days after the patient’s visit. *L. pneumophila* was isolated from these samples, yielding 60, 57, 22, and 1 isolate, respectively, for a total of 140 isolates (Table S1). Of these, 138 isolates belonged to serogroup 1 (SG1) and were all sequence type (ST) 138. The remaining two isolates belonged to serogroup 5 (SG5) and were both ST1631, recovered from surface swab samples collected from bathtubs A and B, respectively.

### cgSNV analysis

Initially, cgSNV analysis was performed on all 140 isolates, including 138 SG1 isolates and 2 SG5 isolates. The complete genome sequence of KLH309, a representative clinical isolate obtained from the patient in the present investigation, was used as the reference. SG1 and SG5 formed clearly separated clusters in the cgSNV network, with 71,355 cgSNVs between the closest nodes connecting the two clusters (Fig. S1). On this basis, the SG5 isolates were considered unlikely to be associated with the investigated case and were excluded from subsequent analyses.

**Fig 1:**
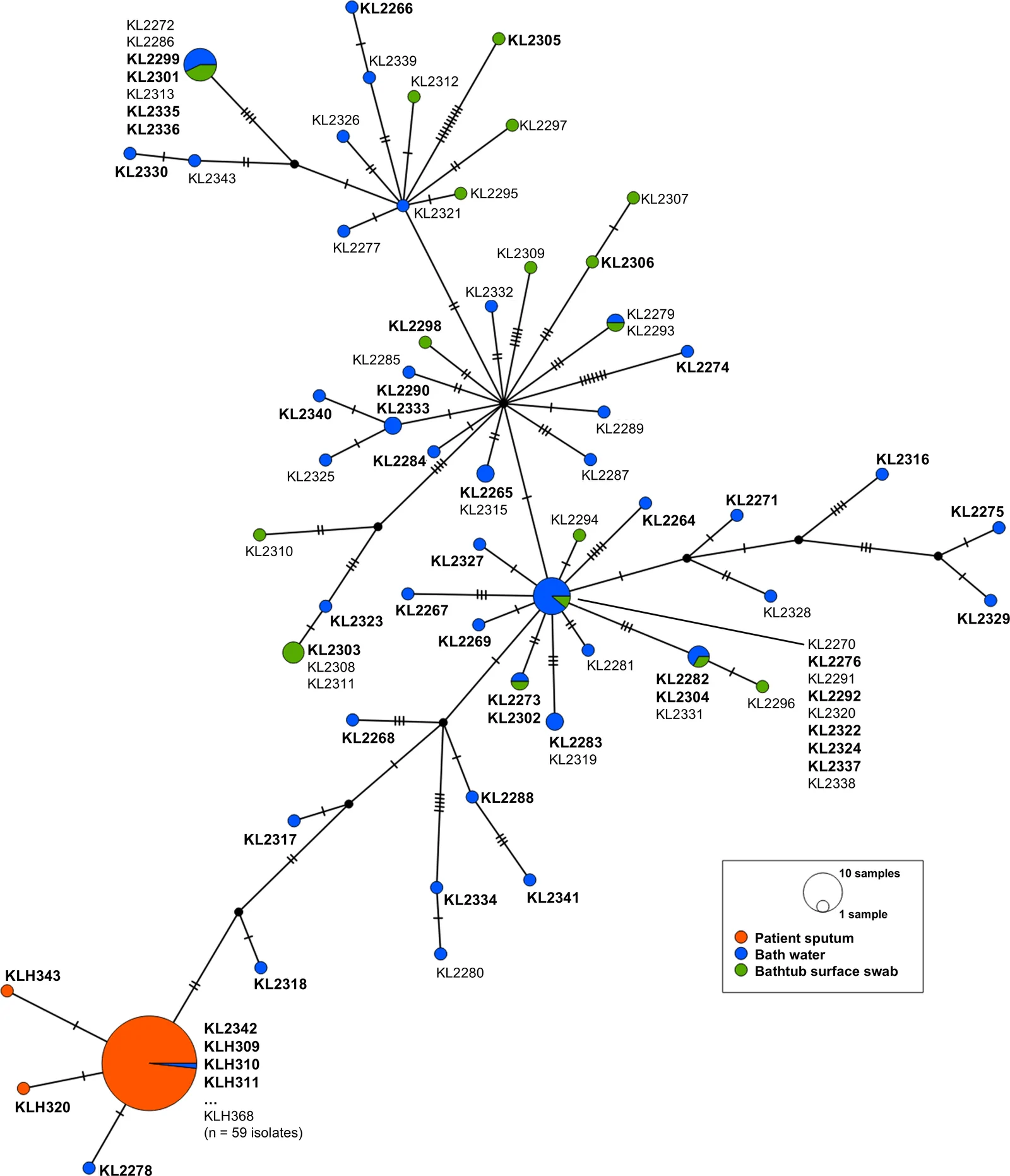
Core genome single-nucleotide variant (cgSNV) network of 138 serogroup 1 isolates. Each node represents a unique cgSNV genotype, and node size is proportional to the number of isolates sharing that genotype. Colors indicate the source of isolation: patient sputum (orange), bathwater (blue), and bathtub surface swab (green). All environmental isolates shown here were obtained from bathtub A. Edges connect nodes, and the number of tick marks on each edge represents the cgSNV distance between genotypes. Isolate labels shown in bold indicate isolates for which complete genome sequences were obtained. The largest cluster consisted of 59 isolates, predominantly from patient sputum, and formed the core of the 62-isolate major clinical lineage analyzed further in this study.

Next, a total of 138 SG1 isolates were analyzed using KLH309 as the reference genome. The core genome comprised 3,188,069 bp, within which 136 cgSNVs were identified. Gubbins’ analysis did not identify any putative recombination regions. The maximum pairwise distance was 20 SNVs, indicating that all isolates were closely related (Fig. 1). The largest node in the cgSNV network contained 59 isolates, including 58 patient sputum isolates and one bathwater isolate (KL2342). Two additional patient isolates (KLH320 and KLH343) and one bathwater isolate (KL2278) were connected to this node by a single SNV. In contrast, most of the remaining environmental isolates (bathwater and surface swabs from bathtub A) were distributed across 52 distinct nodes comprising 76 isolates, consistent with considerable fine-scale genetic diversity among environmental isolates.

To further characterize the major clinical lineage, the 59-isolate node in the cgSNV network (Fig. 1), together with three closely related isolates differing by one SNV, was analyzed collectively as a 62-isolate major lineage. As a result, only two shared SNV positions (2956577 and 3126001, KLH309 reference coordinates) were identified (Table S2). Both SNVs were located within coding sequences (CDSs) and resulted in non-synonymous substitutions in a Dot/Icm type IV secretion system effector (LegS1) and a heme biosynthesis HemY-related protein.

### Complete genome sequencing

To investigate genome structural differences beyond cgSNV-level nucleotide variation, complete genome sequences were obtained for 48 representative isolates. All assemblies were circularized, indicating fully resolved genomes (Table S3). Genome sizes ranged from 3,401,904 to 3,423,096 bp, with 3,015–3,035 predicted CDSs per genome. The number of rRNA and tRNA genes was identical across all isolates.

### Genome structural variation

**Fig 2:**
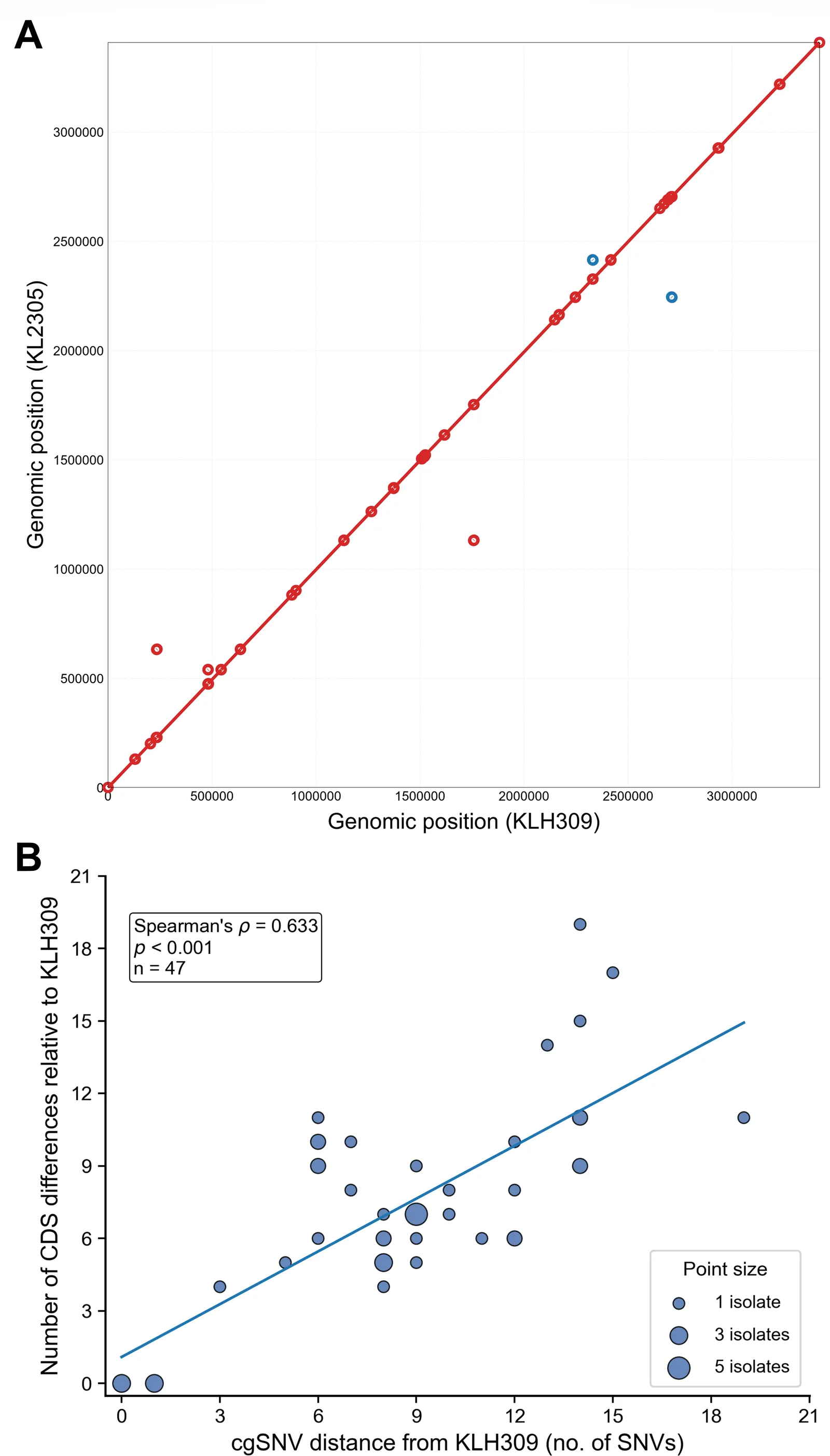
Genome-wide structural conservation and relationship between cgSNV distance and CDS differences among isolates. (**A**) Whole-genome alignment between KLH309 and the representative isolate KL2305, selected as the most divergent isolate based on cgSNV distance. Genomic positions of KLH309 (*x*-axis) are plotted against those of KL2305 (*y*-axis). Each point represents a homologous region identified by sequence alignment. Forward and reverse alignments are shown in red and blue, respectively. The continuous diagonal alignment indicates that gene order is largely conserved across isolates. (**B**) Relationship between cgSNV distance from KLH309 and the number of CDS differences relative to KLH309 among non-reference complete-genome isolates (*n* = 47). Each point represents an isolate, and point size reflects the number of isolates sharing the same cgSNV distance and CDS difference values. A positive correlation was observed between cgSNV distance and CDS differences (Spearman’s *ρ* = 0.633, *P* < 0.001).

Using these complete genome sequences, we examined whether differences existed in gene order and gene content among the isolates, using strain KLH309 as the reference. Comparative genome analysis revealed that all isolates retained nearly identical gene synteny relative to KLH309 (Fig. 2A). Dot plot analysis showed continuous diagonal alignments, indicating that no large-scale genomic rearrangements, such as major inversions, insertions, or deletions, were observed.

BLAST-based comparison of CDSs showed that the number of CDS differences relative to KLH309 was at most 19 per isolate (Fig. 2B). Most of these differences were classified as fragment-like CDSs, accounting for 331 of 342 sites (96.8%), and exhibited high sequence identity (98.0%–100.0%) but reduced query coverage (32.0–89.9%), suggesting that many CDSs were fragmented or only partially conserved rather than completely absent (Table S4). These differences were distributed across multiple genomic loci, whereas CDSs classified as absent (no hit) were limited in number, occurring as a single CDS in KL2276 and as five consecutive CDSs only in KL2303 and KL2323. Furthermore, a significant positive correlation was observed between the cgSNV distance from KLH309 and the number of CDS differences across isolates (Spearman’s *ρ* = 0.633, *P* < 0.001), indicating that CDS variation increased with genetic distance.

To further evaluate the contribution of CDS differences to the inferred genetic relationships, a median-joining network based on cgSNVs alone was constructed using the 48 isolates with complete genome sequences (Fig. S2A). A binary matrix representing 100 variable KLH309 locus tags showing CDS differences (Table S4) was incorporated into the cgSNV alignment to generate a second median-joining network (Fig. S2B). The overall network topology remained largely unchanged after incorporation of CDS differences. However, several isolates that shared identical or closely related nodes in the cgSNV-based network were resolved into distinct nodes in the combined network. For example, five isolates (KL2276, KL2292, KL2322, KL2324, and KL2337), which formed a single node in the cgSNV-based network, were resolved into distinct nodes after incorporation of CDS differences.

### Diversity of IS insertion patterns across isolates

Using complete genome sequences, we investigated the presence and genomic distribution of ISs. IS insertion sites were represented as “anchors” defined by the combination of flanking genes surrounding each insertion locus. Across the 48 isolates, up to 12 IS families were identified, collectively distributed across 190 union anchor loci (Table S5). These union anchor loci were subsequently mapped onto the KLH309 reference genome, resulting in 170 distinct reference anchor loci (Table S6).

**Fig 3:**
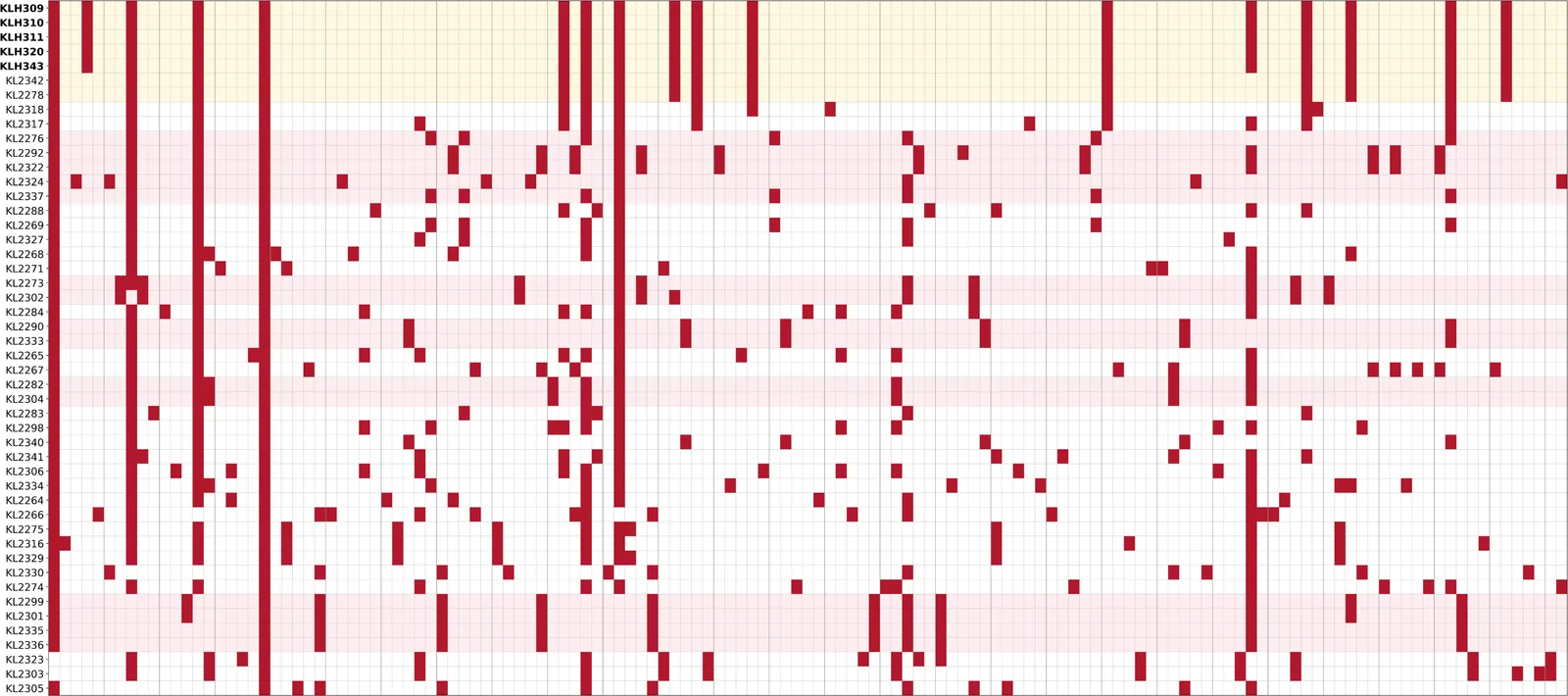
Distribution of IS256 insertion sites across isolates. Each row represents an individual isolate, and each column represents an IS256 reference anchor locus arranged from left to right according to its reference anchor position, which corresponds to the genomic order on the KLH309 reference genome. Red cells indicate the presence of IS256 at the corresponding locus, whereas blank cells indicate absence. Multiple IS copies sharing the same reference anchor locus are represented as a single column in the matrix. Isolates are arranged according to increasing cgSNV distance from KLH309. Pale pink backgrounds denote isolates belonging to the same cgSNV node. A subset of isolates included in the 62-isolate lineage—defined as the largest cgSNV node together with three closely related isolates differing by one SNV (KLH320, KLH343, and KL2278)—is highlighted in pale yellow.

Among these, IS256 was present in all isolates (48/48, 100.0%) and exhibited the greatest diversity in genomic distribution. IS256 insertions were detected at 137 distinct reference anchor loci, indicating broad diversity of insertion sites across the genome. Each isolate harbored 11–20 copies located at 10–17 distinct reference anchor loci (Table 1; Fig. 3). The mean number of isolates sharing each IS256 reference anchor was 4.8, indicating that most insertion sites were shared by only a limited number of isolates. As many as three IS256 copies were detected at a single reference anchor. For example, at reference anchor RA00017 in KL2342, three IS256 copies were arranged in close proximity within the corresponding anchor region (Table S6). At the sequence level, the adopted IS256 elements were 1,409 or 1,411 bp in length. The 1,411-bp elements shared an identical 1,409-bp internal sequence with the 1,409-bp elements and differed only by one additional nucleotide at each end, indicating an alternative boundary prediction rather than sequence variation.

**TABLE 1:** Distribution and insertion site diversity of insertion sequence (IS) families across complete-genome isolates

IS family	Positive isolates (%)	Total IS copies	Copy number per isolate	Distinct reference anchor loci	Distinct reference anchor loci per isolate	Mean no. of isolates sharing each reference anchor
IS256	48/48 (100.0)	707	11–20	137	10–17	4.8
IS982	47/48 (97.9)	106	0–3	7	0–3	15.1
IS4	48/48 (100.0)	200	4–5	5	4–5	40.0
IS110	48/48 (100.0)	113	2–4	5	2–4	22.6
IS21	9/48 (18.8)	9	0–1	4	0–1	2.3
ISL3	48/48 (100.0)	97	2–3	3	2–3	32.3
ISNCY	48/48 (100.0)	49	1–2	3	1–2	16.3
IS3	48/48 (100.0)	95	1–2	2	1–2	47.5
IS701	47/48 (97.9)	47	0–1	1	0–1	47.0
IS5	48/48 (100.0)	48	1–1	1	1–1	48.0
ISKra4	48/48 (100.0)	48	1–1	1	1–1	48.0
IS630	45/48 (93.8)	45	0–1	1	0–1	45.0

Among less prevalent IS families, IS21 was detected in only nine isolates (18.8%) but was distributed across four reference anchor loci (Table 1; Fig. S3). The mean number of isolates sharing each IS21 reference anchor was 2.3, indicating that these insertions were restricted to a subset of isolates and suggesting lineage-specific insertion events. The remaining 10 IS families were widely distributed across isolates, being present in 93.8%–100.0% of isolates. In contrast to IS256, these ISs exhibited low diversity in insertion sites, being distributed across a limited number of reference anchor loci (*n* = 1–7), and high sharing across isolates, with a mean of 15.1–48.0 isolates sharing each reference anchor. This pattern suggests that these ISs are largely fixed within the population, with conserved insertion sites across isolates.

Notably, differences in IS insertion patterns were observed even among isolates sharing identical cgSNV genotypes (i.e., within the same node in the cgSNV network; Fig. 3 and Fig. S3), indicating that IS-mediated structural variation can occur independently of core genome SNV variation. Among the seven complete-genome isolates representing the 62-isolate major lineage shown in Fig. 1, 34 of the 36 detected reference anchor loci were shared by all isolates (Table S6). The remaining two loci (RA00006 and RA00136), both corresponding to IS256, were detected only in the five clinical isolates. Overall, these isolates exhibited relatively similar IS distribution patterns, suggesting that only a subset of the structurally diverse environmental population was associated with infection.

### Association of IS with CDS fragmentation

To investigate whether ISs contribute to the fragmentation of the CDS differences identified above relative to the reference strain KLH309, we examined the positional relationship between ISs and CDS differences in each isolate. Across all isolates, a total of 342 CDS difference sites were identified, and, for each isolate, the genomic positions of these CDS differences were compared with those of IS insertions identified in the same genome. As a result, 198 sites (57.9%) were found to overlap with or be located within 10 bp of an IS, suggesting an association between IS insertion and CDS fragmentation (Table 2; Table S4). The number of IS-associated CDS differences varied across isolates (Table S7). Among these IS-associated sites, IS256 was predominant, accounting for 191 cases (96.5%). The affected CDSs encompassed a wide range of genes, including hypothetical proteins and genes involved in diverse cellular functions, with no apparent functional enrichment (Table S4).

**TABLE 2:** Association of insertion sequences (ISs) with CDS differences among complete-genome isolates^*a*^

CDS difference category	Subcategory	Count	Percentage (%)
IS associated		198	57.9
	IS256	191	96.5
	IS21	4	2.0
	IS982	2	1.0
	ISNCY	1	0.5
Non-IS associated		144	42.1

^*a*^ Counts and proportions of CDS differences associated with IS insertions are shown. Percentages for individual IS families were calculated among IS-associated CDS differences.

### *G. mellonella* infection assay

To assess whether representative clinical and environmental isolates differed in virulence, a *G. mellonella* infection assay was performed using six isolates selected based on the cgSNV network analysis: two clinical isolates (KLH309 and KLH320), two environmental isolates closely related to clinical isolates (KL2318 and KL2342), and two environmental isolates representing genetically distinct nodes containing multiple isolates (KL2276 and KL2335). Three isolates (KLH309, KLH320, and KL2342) carried the reference allele combination at the two shared non-synonymous SNV positions identified in the major clinical lineage, whereas the remaining three isolates (KL2318, KL2276, and KL2335) carried the alternative allele combination. All isolates caused progressive reductions in larval health index scores over time, and similar temporal patterns were observed across isolates (Fig. S4). No significant differences in health index scores were detected among isolates at any evaluated time point (Kruskal–Wallis test, all *P* > 0.05). In contrast, PBS-injected and non-injected control larvae showed no apparent reduction in health index scores throughout the experimental period.

## DISCUSSION

In this study, we investigated the genomic diversity of *L. pneumophila* isolates associated with human infection by integrating cgSNV analysis with comparative analysis of genome structure using complete genome sequences, with a particular focus on structural variation beyond cgSNVs. By analyzing multiple isolates from each sample, we sought to capture within-source genetic diversity at high resolution.

cgSNV analysis demonstrated that the clinical isolates were highly clonal, with 58 isolates forming an identical node and two isolates differing by only one SNV, indicating infection by a single clonal lineage. In contrast, environmental isolates from the same bathtub exhibited fine-scale genetic diversity, comprising 52 distinct cgSNV genotypes despite a maximum pairwise distance of only 20 SNVs. Similar coexistence of multiple genetic subtypes within a single bath facility has been reported previously in *Legionella* outbreak investigations (13, 15). Despite this diversity, the isolates in our study remained closely genetically related, differing by no more than 20 cgSNVs, consistent with ongoing microevolution within a localized environmental reservoir.

Only two environmental isolates were identical or closely related (within one SNV) to the major clinical lineage. These findings indicate that, in this investigation, cgSNV-based analysis was useful for identifying the environmental lineage most closely related to the clinical isolates. The major clinical lineage differed from the most closely related environmental isolates by only 2 shared non-synonymous SNVs, while all isolates in the population differed by no more than 20 cgSNVs. Given this limited nucleotide variation, we investigated whether additional genomic differences not captured by cgSNV analysis could be identified through complete genome comparison of representative isolates. Whole-genome alignment of complete genome sequences revealed highly conserved genome synteny across isolates, whereas BLAST-based CDS comparisons showed that most CDS differences exhibited high sequence identity but reduced query coverage and were classified as fragment-like. Together, these results suggest that, within this closely related population, genomic differences were more often associated with localized gene disruption than with large-scale genomic change. Incorporation of these CDS differences into the cgSNV-based network preserved the overall topology while resolving several isolates that shared the same cgSNV node, suggesting that CDS-level structural variation complements cgSNV-based analysis in closely related populations.

Further analysis of ISs revealed distinct distribution patterns across isolates, with IS256 exhibiting particularly high diversity in insertion sites. Among the 12 identified IS families, IS256 was present in all isolates, with insertions detected at 137 distinct reference anchor loci. Each isolate harbored numerous unique insertion loci with limited sharing, suggesting extensive diversification associated with IS256 transposition events. In contrast, most other IS families showed conserved insertion patterns across isolates, suggesting limited mobility. The high mobility of IS256 has been reported in other bacterial species, such as *Staphylococcus aureus*, where it contributes to genomic diversification and adaptation (23), supporting a similar role in *L. pneumophila* populations. A recent experimental evolution study in *Escherichia coli* further demonstrated that stable, nutrient-rich conditions under relaxed selection can promote extensive IS-mediated genome structural evolution (24). Such conditions may partially resemble the relatively stable and nutrient-rich environment within bath systems and may have contributed to the accumulation of IS-mediated structural variation within localized environmental reservoirs.

Despite the highly clonal population structure defined by cgSNV analysis, variation in IS insertion patterns was observed even among isolates sharing identical cgSNV genotypes, indicating that IS-mediated structural variation can occur independently of core genome SNV accumulation. Accordingly, mobile IS elements, particularly IS256 and, to a lesser extent, IS21 and related families, may provide complementary markers for resolving fine-scale relationships among closely related isolates with minimal SNV differences.

To assess the contribution of IS insertions to CDS fragmentation, CDS difference sites were compared with IS insertion loci, revealing that more than half of the CDS differences relative to the reference strain were located at or near IS insertion sites and that most of these were associated with IS256. In addition, the affected genes were functionally diverse and showed no clear enrichment. Together, these findings suggest that IS-mediated disruption contributed substantially to CDS fragmentation within the population and occurred broadly across the genome rather than targeting specific functional pathways.

Despite this extensive IS-associated genomic diversification, the major clinical lineage exhibited relatively similar IS insertion patterns, and infection model experiments likewise did not reveal clear differences in virulence among representative clinical and environmental isolates. Environmental samples were collected approximately 13 days after the patient’s use of the bathing facility. Multiple cgSNV-defined genotypes coexisted within the same environmental source, whereas only a subset was recovered from the patient. These observations suggest that the fine-scale genetic diversity observed among the environmental isolates was already present at the time of exposure. This interpretation supports the possibility that the clinical lineage originated from the diverse environmental population through opportunistic exposure rather than through selection of a uniquely virulent genotype, a concept that has been proposed in previous studies of *Legionella* infection (7).

Our strategy of collecting multiple isolates from individual samples revealed that the clinically associated lineage represented only a subset of the genomic diversity present within a single environmental source, underscoring the importance of sufficient sampling density for accurately characterizing environmental populations. Furthermore, IS profiling revealed additional fine-scale structural diversification even among isolates sharing identical cgSNV genotypes. In this study, these approaches provided a higher-resolution view of within-source genomic diversity than would have been achievable through conventional analyses based on a single or limited number of isolates.

This study has several limitations. First, our analysis was based solely on genomic sequence data, and the functional consequences of CDS fragmentation and IS insertion were not directly evaluated at the transcriptional or phenotypic level. In addition, ongoing IS256 transposition activity was not investigated. Second, environmental sampling was conducted at a single time point, which may not fully capture temporal changes in genomic diversity within the source population. Third, this study was based on isolates from a single epidemiological setting, and further studies across multiple settings and environmental conditions will be required to generalize these findings and to determine whether additional mobile IS elements beyond IS256 and IS21 contribute to genomic structural variation. Future studies incorporating transcriptomic or functional assays, as well as longitudinal and multi-site sampling, will be necessary to determine the biological and epidemiological significance of these structural variations.

Overall, our findings highlight the importance of integrating population-level sampling and structural genome analysis to better resolve genomic diversity within infection-associated environmental reservoirs of *L. pneumophila*.

## MATERIALS AND METHODS

### Isolation and identification of bacterial strains

This study was conducted as part of an epidemiological investigation of a legionellosis case that occurred in 2024, in which isolates were obtained from both a patient and a suspected environmental source associated with the same exposure setting. To capture within-source genomic diversity, extensive sampling was performed, and a large number of colonies were isolated from each sample for downstream genomic analysis.

Bacterial isolates were obtained from patient sputum and environmental samples, including bathwater and surface swabs from the bathing facility used by the patient, with one sample collected from each sampling source. Samples were collected within a similar time frame and cultured on MWY and GVPCα agar at 37°C for at least 3 days. Individual *Legionella*-like colonies grown on these media were subcultured on BCYEα agar for purification, and each pure culture derived from a single colony was treated as one isolate. For samples yielding fewer than 60 colonies, all available colonies were collected, whereas for samples yielding more than 60 colonies, up to 60 colonies were selected to maximize the representation of within-sample diversity. Isolates were identified as *L. pneumophila* by species-specific PCR (25) and serogrouped by slide agglutination.

### Whole-genome sequencing and read processing

Genomic DNA was extracted from all isolates using a modified protocol based on the method described by Murase et al. (26). Briefly, a loopful of bacterial colonies grown on BCYEα agar at 37°C for approximately 48 h was suspended in a mixture of 0.1 M NaCl/TE buffer and chloroform containing 0.2 mm diameter glass beads. Cells were disrupted using a bead beater (Yasui Kikai Corporation, Osaka, Japan) at 3,000 rpm for 1 min. The lysate was treated with RNase A, followed by purification using the phenol-chloroform extraction method. DNA was precipitated with isopropanol and resuspended in EB buffer (QIAGEN, Hilden, Germany).

For short-read sequencing, libraries were prepared from extracted DNA using the QIAseq FX DNA Library Kit (QIAGEN) according to the manufacturer’s instructions. Sequencing was performed by a commercial provider (Rhelixa Inc., Tokyo, Japan) using the NovaSeq X platform (Illumina, San Diego, CA, USA) to generate 150-bp paired-end reads. Raw reads were processed using fastp v1.1.0 (27), and the resulting reads were used for downstream analyses.

For long-read sequencing, libraries were prepared using the Rapid Barcoding Kit (Oxford Nanopore Technologies, Oxford, United Kingdom) and sequenced on a MinION Mk1B device (Oxford Nanopore Technologies). Basecalling was performed using Dorado v1.0.2, and raw reads were processed using NanoFilt v2.8.0 (28). Reads shorter than 1,000 bp or with a mean quality score below Q10 were removed. The filtered reads were used for subsequent analyses.

### Sequence-based typing

STs of all isolates were determined using the sequence-based typing (SBT) allele database of the European Working Group for Legionella Infections (EWGLI) (29, 30). The seven SBT loci (*flaA*, *pilE*, *asd*, *mip*, *mompS*, *proA*, and *neuA*) were identified from contigs assembled from short-read data using Unicycler v0.5.1 (31) by comparison against the EWGLI SBT allele database using BLASTN (32). For each locus, the best hit was selected based on sequence identity and alignment coverage, and the corresponding allele number was assigned. The combination of allele numbers across the seven loci was used to determine the ST of each isolate.

### cgSNV analysis

SNVs were identified for all isolates based on short-read data using the SNPcaster pipeline v0.9.5 (https://github.com/leech-rr/SNPcaster), which integrates BactSNP (https://github.com/IEkAdN/BactSNP), Snippy (https://github.com/tseemann/snippy), and recombination analysis using Gubbins (33). The complete genome sequence of strain KLH309 was used as the reference because it was a representative clinical isolate with a fully assembled genome.

Initial SNV calling and pseudo-genome generation were performed using BactSNP with an allele frequency threshold of 0.9. Core genome SNVs were subsequently extracted using Snippy with default parameters, requiring a minimum read depth of 10. SNVs located in repetitive regions were excluded using the SNPcaster pipeline. Only SNVs located in the core genome shared among all isolates were retained. Recombination analysis was performed within the SNPcaster pipeline using Gubbins. No clustered SNV removal or masking was applied. The resulting core cgSNV alignment was used to construct a median-joining haplotype network using PopART (34).

### Selection of isolates for complete genome sequencing and comparative analysis

To investigate genomic structural variation beyond core genome SNVs, including insertion sequence dynamics, 48 representative isolates were selected based on the cgSNV network structure. Complete genome sequences were obtained for these isolates and used for comparative genomic analysis. Selection criteria included coverage of the major cluster and its closely related isolates, as well as genetically diverse environmental isolates distributed across multiple nodes in the cgSNV network, while ensuring representation of different isolation sources.

### Genome assembly and annotation

To generate complete genome sequences, hybrid assembly was performed using Hybracter v0.12.0 (https://github.com/gbouras13/hybracter) with both short- and long-read data. The resulting assemblies were assessed for circularity using Circlator v1.5.5 (35) and for genome completeness and contamination using CheckM2 v1.1.0 (36). Genome annotation was performed using DFAST with default parameters (37). To ensure consistency across genomes, sequences were rotated so that the *dnaA* gene was positioned approximately 100 bp downstream of the origin of replication. The obtained complete genome sequences and corresponding annotated GenBank files were used for downstream analyses.

### Comparative genomic structural analysis and CDS-based comparison

Genomic structural variation among isolates was assessed using complete genome sequences. Whole-genome alignments were performed against the reference strain KLH309 using the nucmer module of MUMmer4 (38), followed by filtering for one-to-one alignments with delta-filter. Dot plots were generated using mummerplot and visualized with gnuplot to evaluate genome-wide synteny between KLH309 and each isolate.

For gene content comparison, CDSs annotated in the KLH309 genome were used as the reference CDS set. CDSs shorter than 90 bp and those associated with IS-related functions (e.g., transposases and recombinases) were excluded. In addition, the *rtxA* gene was excluded because substantial strain-dependent length variation of this gene has been reported previously (39), which could confound the interpretation of CDS fragmentation. Each reference CDS was compared against all isolates using BLASTN, and the best hit was selected based on alignment score. A CDS was considered “present” when the sequence identity was ≥90% and query coverage was ≥90%. CDSs not meeting these criteria were classified as “fragment-like” (identity ≥90% but coverage <90%), “partial hit” (identity <90%), or “no hit” (no detectable hit). The number of CDS differences for each isolate relative to KLH309 was defined as the total number of CDSs classified as fragment-like, partial hit, or no hit.

To assess the relationship between nucleotide-level variation and gene-level differences, cgSNV distances from KLH309 were obtained from the SNV matrix generated by SNPcaster. The number of CDS differences per isolate was plotted against the cgSNV distance, and the correlation was evaluated using Spearman’s rank correlation coefficient. Visualization was performed using Python (matplotlib), and duplicated data points were represented as bubble plots proportional to the number of isolates sharing the same coordinates.

To evaluate the contribution of CDS differences to the inferred genetic relationships among the 48 isolates with complete genome sequences, CDS differences identified by the BLAST-based comparison described above and summarized in Table S4 were converted into a binary character matrix. Each variable KLH309 locus tag was treated as a single binary character, with fragment-like or no-hit CDSs scored as 1 and isolates without CDS differences scored as 0. The binary character matrix was appended to the cgSNV alignment of the corresponding 48 isolates, and a median-joining network was generated.

### Identification of insertion sequences and definition of anchor loci

ISs were identified from complete genome assemblies using ISEScan v1.7.3 (40). IS candidates were retained when they met the following criteria: the presence of an open reading frame and terminal inverted repeats, and a sequence length between 700 and 3,000 bp. When predicted IS elements assigned to different IS families overlapped, all IS predictions involved in the overlap were excluded from the IS data set used in this study. Candidates located near the beginning of contigs and overlapping putative *dnaA*-associated regions were excluded as likely artifacts. Only ISs that met these criteria (“adopted ISs”) were used for downstream analyses. For the primary insertion-site analysis, IS elements were classified according to the IS family assignments generated by ISEScan. Because IS256 exhibited the greatest diversity in genomic distribution and was the focus of the detailed insertion-site analysis, the full-length nucleotide sequences of the adopted IS256 elements were aligned using MAFFT v7.526 (41) and subsequently compared by pairwise BLASTN to assess sequence variation.

For each adopted IS, its genomic position was compared with CDS annotations based on DFAST-annotated GenBank files. Overlapping CDSs were identified, and the CDS most closely associated with each IS was selected based on overlap length to define the local genomic span encompassing the IS-associated CDS region.

Flanking genes were then identified based on RefSeq protein identifiers (WP IDs) relative to this genomic span. Only CDSs annotated with WP IDs and not related to IS elements (e.g., transposases or recombinases) were considered. For each IS locus, up to two upstream and two downstream flanking CDSs were selected, prioritizing genes closest to the boundaries of the defined span. These four genes (left2, left1, right1, and right2) were used to define a genomic anchor, representing the insertion context and enabling comparison of IS insertion positions across isolates.

### Integration and reference mapping of anchor loci

To enable isolate-to-isolate comparison of IS insertion positions, anchors were defined independently for each isolate and subsequently integrated across all isolates to construct a non-redundant set of anchor loci. Across isolates, isolate-specific anchors sharing the same IS family and the same combination of WP IDs for the two nearest CDSs on each side of the IS were assigned to the same union anchor, thereby generating a non-redundant set that included all distinct IS insertion loci identified in at least one isolate. Each union anchor was then assigned a unique identifier (union anchor ID; e.g., UA00001).

To determine the genomic positions of union anchor loci on the reference genome, a reference anchor framework was constructed using the complete genome of strain KLH309. After excluding CDSs constituting IS elements from the KLH309 genome, the remaining CDSs with WP IDs were ordered along the genome, and consecutive groups of four genes were used to generate a reference set of outer four-gene combinations.

To determine the virtual position of each union anchor within the KLH309 reference framework, each union anchor was mapped to the framework as follows. For each union anchor, mapping was first attempted using all four flanking WP IDs. If no four-ID match was found, mapping was subsequently attempted using three WP IDs and then two WP IDs. For three-ID mapping, all eight possible ordered three-ID matching patterns were evaluated by requiring the two WP IDs immediately adjacent to the IS on either the left or right side while allowing either of the two WP IDs on the opposite side to match. For example, a union anchor with the arrangement A-B-IS-C-D was evaluated using the patterns A-B-IS-C-*, A-B-IS-*-D, A-B-IS-D-*, A-B-IS-*-C, A-*-IS-C-D, *-B-IS-C-D, B-*-IS-C-D, and *-A-IS-C-D, where * denotes a wildcard that may correspond to any WP ID. The wildcard is used here only to illustrate the matching procedure and was not included as an actual value in the analysis. For two-ID mapping, either the two WP IDs immediately to the left of the IS or the two WP IDs immediately to its right were used. If a union anchor matched multiple reference anchors at the same matching level, the reference anchor located furthest upstream in the KLH309 reference framework was selected. Union anchors mapped to the same position in the reference framework and associated with the same IS type were grouped into a single reference anchor, irrespective of the matching level. Each reference anchor was then assigned a unique identifier (reference anchor ID; e.g., RA00001). For each reference anchor, the number of isolates sharing that locus was calculated, and the mean number of isolates sharing each reference anchor was calculated for each IS family. The positions of the reference anchors were represented by the locus numbers of the third WP IDs in their corresponding four-WP-ID combinations in the KLH309 genome.

### Assessment of the positional relationship between CDS differences and ISs

To evaluate whether ISs are associated with CDS fragmentation observed in each isolate, the positional relationship between CDS difference sites and adopted ISs was examined within individual sample genomes. CDS differences were defined as described above based on BLAST comparisons using KLH309 CDSs as queries. For each CDS difference, the genomic coordinates on the sample genome were obtained from the best BLAST hit on the sample genome, specifically using the start and end positions of the aligned region.

Adopted IS coordinates for each sample were obtained from the IS detection analysis described above. For each CDS difference site, the nearest IS within the same sample genome was identified based on genomic distance. The distance between a CDS and an IS was defined as the minimum distance between their genomic intervals, with overlapping regions assigned a distance of zero.

A CDS difference was classified as IS associated if it overlapped with an IS or was located within 10 bp of an IS; otherwise, it was classified as not IS associated. For each CDS difference, the IS family of the associated or nearest IS and the distance to the nearest IS were recorded. Summary statistics, including the total number of IS-associated CDS differences and their distribution across IS families, were calculated across all isolates.

### *G. mellonella* infection assay

The *G. mellonella* infection assay was performed based on the method described by Sousa et al. (7) with minor modifications. A preliminary experiment was conducted to determine the appropriate inoculum size. Representative clinical and environmental isolates, KLH309 and KL2276, were selected. Colonies cultured for 48 h were suspended in 1/50 M PBS and adjusted to concentrations of 10^7^–10^9^ CFU/mL. Bacterial concentrations were confirmed by plating serial 10-fold dilutions onto BCYEα agar and counting colonies after 3 days of incubation. A volume of 10 μL of each suspension was injected into larvae, corresponding to approximately 10^5^–10^7^ CFU per larva. Larvae injected with 10 μL of 1/50 M PBS and non-injected larvae were included as controls. In the preliminary experiment, 6 larvae per group were used, whereas 10 larvae per group were used in the main experiment to improve the robustness of the comparisons. Infected larvae were incubated at 37°C and monitored at 18, 24, 48, and 72 h post-infection. Larval health was scored on a 10-point scale according to previously described criteria (42).

Based on the preliminary experiment, an inoculum of approximately 10^6^ CFU per larva was selected, as it produced a measurable level of mortality without causing immediate lethality, allowing comparison of infection dynamics among isolates. Using this condition, six isolates were selected for the main experiment based on the cgSNV network analysis: two clinical isolates (KLH309 and KLH320), two environmental isolates closely related to clinical isolates (KL2318 and KL2342), and two environmental isolates representing genetically distinct nodes containing multiple isolates (KL2276 and KL2335). Each isolate was injected into larvae using the same protocol as described above. Control groups included PBS-injected and non-injected larvae. Larvae were incubated at 37°C and evaluated at the same time points using the same scoring system. The experiment was performed in three independent biological replicates. Actual inoculum sizes determined by viable colony counts ranged from approximately 2.1 × 10^6^ to 3.0 × 10^6^ CFU per larva across all experiments and isolates.

Health index scores were summarized as mean ± standard error of the mean from three independent experiments and plotted against time post-infection. Differences in health index scores among isolates at each evaluated time point were assessed using the Kruskal–Wallis test. A *P* value of <0.05 was considered statistically significant. Statistical analyses and figure generation were performed using Python.

## Data Availability

The available sequencing read data and complete genome sequences generated in this study have been deposited in the DDBJ Sequence Read Archive and DDBJ/GenBank under BioProject accession number PRJDB42229. Individual accession numbers are listed in Tables S1 and S3.

## SUPPLEMENTAL MATERIAL

Supplemental figures aem.01127-26-s0001.pdf Fig. S1 to S4.

Supplemental tables aem.01127-26-s0002.xlsx Tables S1 to S7.

ASM does not own the copyrights to Supplemental Material that may be linked to, or accessed through, an article. The authors have granted ASM a non-exclusive, world-wide license to publish the Supplemental Material files. Please contact the corresponding author directly for reuse.
