# Supplementary material for: Fine-scale structural diversification among closely related Legionella pneumophila isolates from a bath-associated environmental reservoir
Source: Appl Environ Microbiol. 2026 Aug 28;92(9):e01127-26. doi: 10.1128/aem.01127-26 (PMC13599499; doi:10.1128/aem.01127-26)
Supplement: Supplementary file 1 [file aem.01127-26-s0001.pdf]

Supplemental Material

Supplementary Fig. S1. Core genome single nucleotide variant (cgSNV) network analysis of all 140 isolates, including 138 SG1 (serogroup 1) isolates and 2 SG5 (serogroup 5) isolates.

Supplementary Fig. S2. Median-joining networks of the 48 isolates for which complete genome sequences were obtained.

Supplementary Fig. S3. Distribution of adopted insertion sequence (IS) anchor loci across complete-genome isolates.

Supplementary Fig. S4. *Galleria mellonella* health index scores following infection with representative clinical and environmental isolates of *L. pneumophila*.

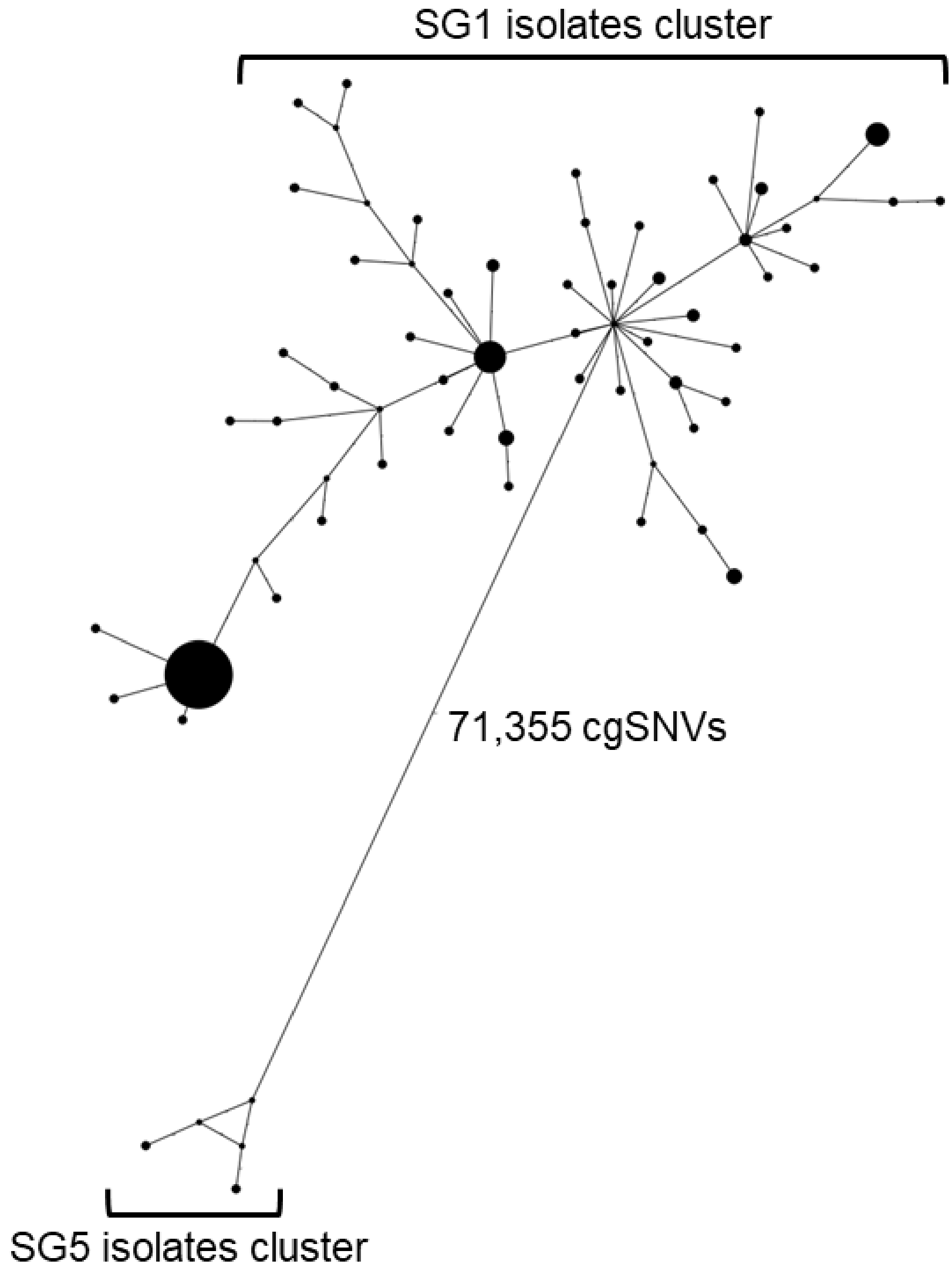

Supplementary Fig. S1. Core genome single nucleotide variant (cgSNV) network analysis of all 140 isolates, including 138 SG1 (serogroup 1) isolates and 2 SG5 (serogroup 5) isolates. SG1 and SG5 formed clearly separated clusters, with a cgSNV distance of 71,355 between the closest nodes connecting the two clusters.

A

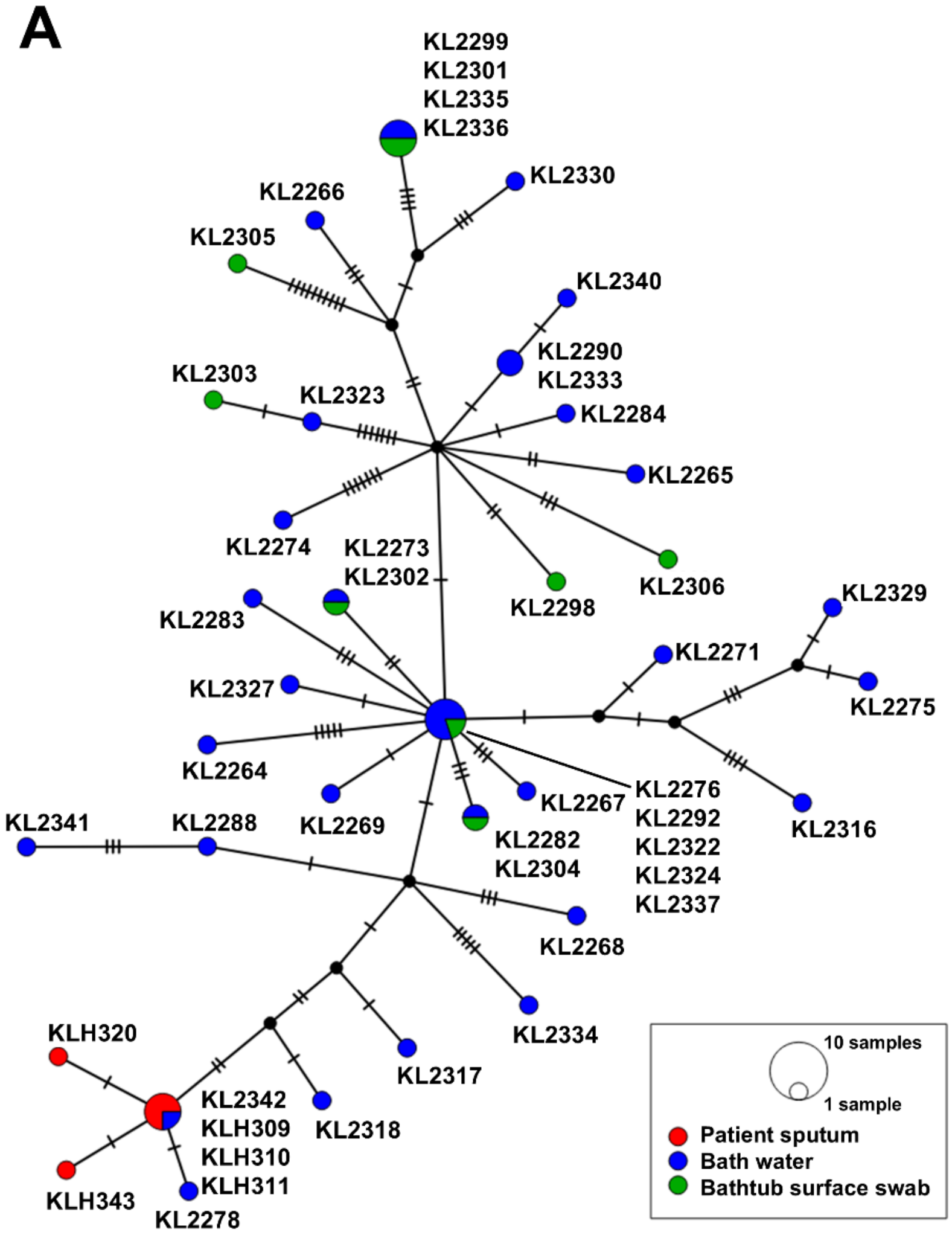
KL2299
KL2301
KL2335
KL2336
KL2330
KL2266
KL2305
KL2340
KL2290
KL2333
KL2303
KL2323
KL2284
KL2265
KL2274
KL2273
KL2302
KL2298
KL2306
KL2329
KL2283
KL2271
KL2275
KL2327
KL2264
KL2276
KL2292
KL2322
KL2324
KL2337
KL2267
KL2269
KL2316
KL2341
KL2288
KL2282
KL2304
KL2268
KL2334
KL2317
KLH320
KL2342
KLH309
KLH310
KLH311
KL2318
KLH343
KL2278
10 samples
1 sample
Patient sputum
Bath water
Bathtub surface swab

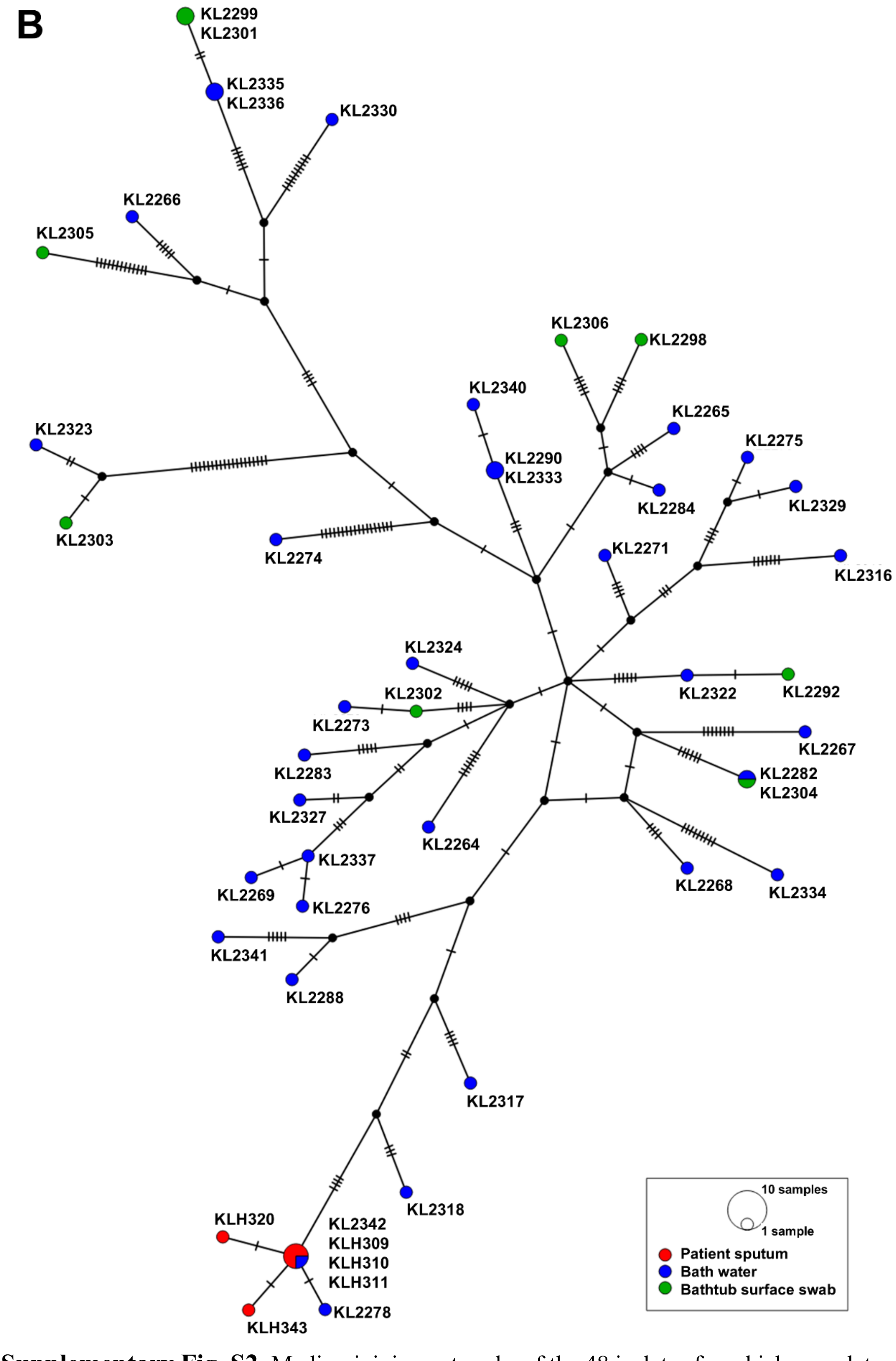

Supplementary Fig. S2. Median-joining networks of the 48 isolates for which complete genome sequences were obtained. (A) Network based on core genome single nucleotide variants (cgSNVs). (B) Network based on the cgSNV alignment supplemented with binary CDS-difference characters derived from the comparative genomic structural analysis (Supplementary Table S4). Each node represents a unique genetic profile, and node size is proportional to the number of isolates sharing that profile. Colors indicate the source of isolation: patient sputum (orange), bath water (blue), and bathtub surface swab (green). All environmental isolates shown here were obtained from Bathtub A. Edges connect nodes, and the number of tick marks on each edge represents the genetic distance between connected nodes. In panel B, the genetic distance includes both cgSNV differences and binary CDS-difference characters.

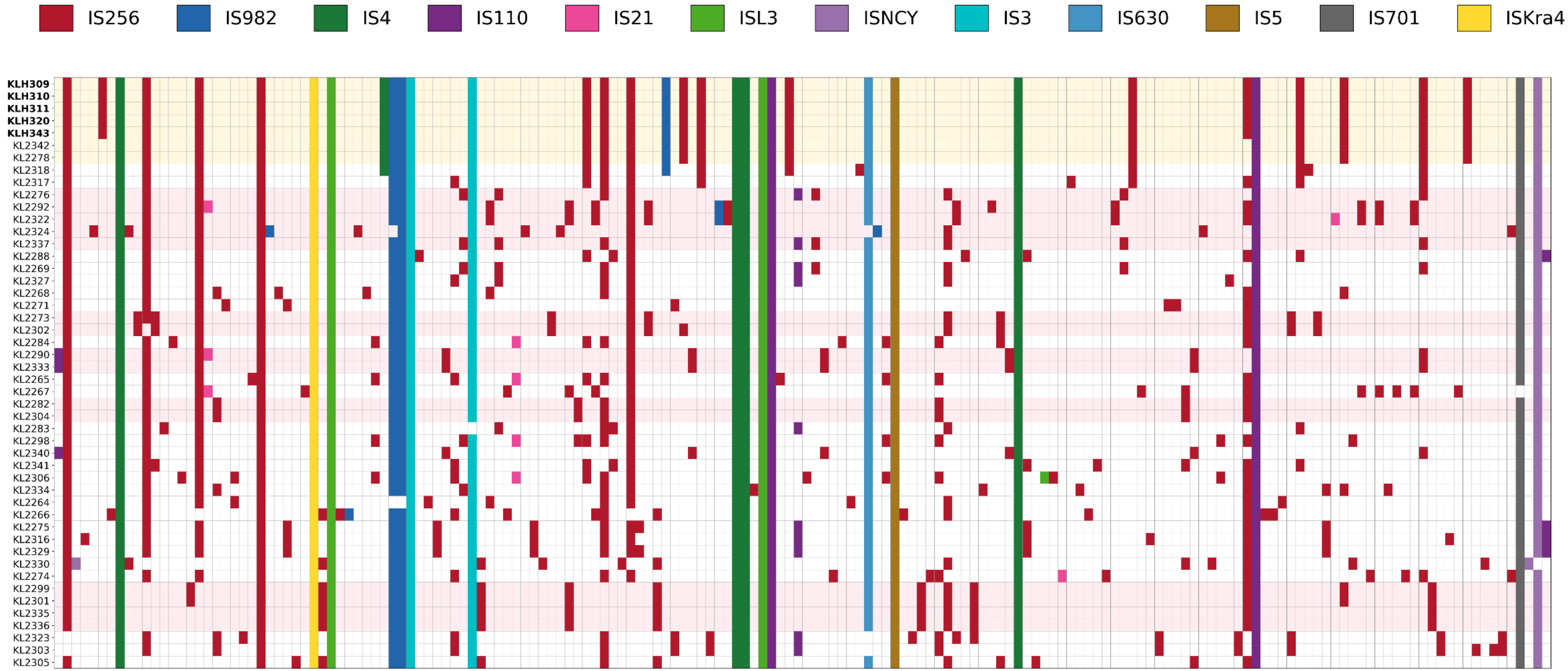

Supplementary Fig. S3. Distribution of adopted insertion sequence (IS) reference anchor loci across complete-genome isolates. Rows represent individual isolates, and columns represent IS reference anchor loci arranged from left to right according to their reference anchor positions, which correspond to the genomic order on the KLH309 reference genome. Colored cells indicate the presence of IS elements at the corresponding loci, with colors representing different IS families. Blank cells indicate absence. Multiple IS copies sharing the same reference anchor locus are represented as a single column in the matrix. Reference anchor loci corresponding to the same reference anchor position but associated with different IS families are displayed as separate columns. Isolates are arranged according to increasing cgSNV distance from KLH309. Pale pink backgrounds denote isolates belonging to the same cgSNV node, and pale yellow backgrounds highlight a subset of isolates included in the 62-isolate lineage defined in Fig. 1.

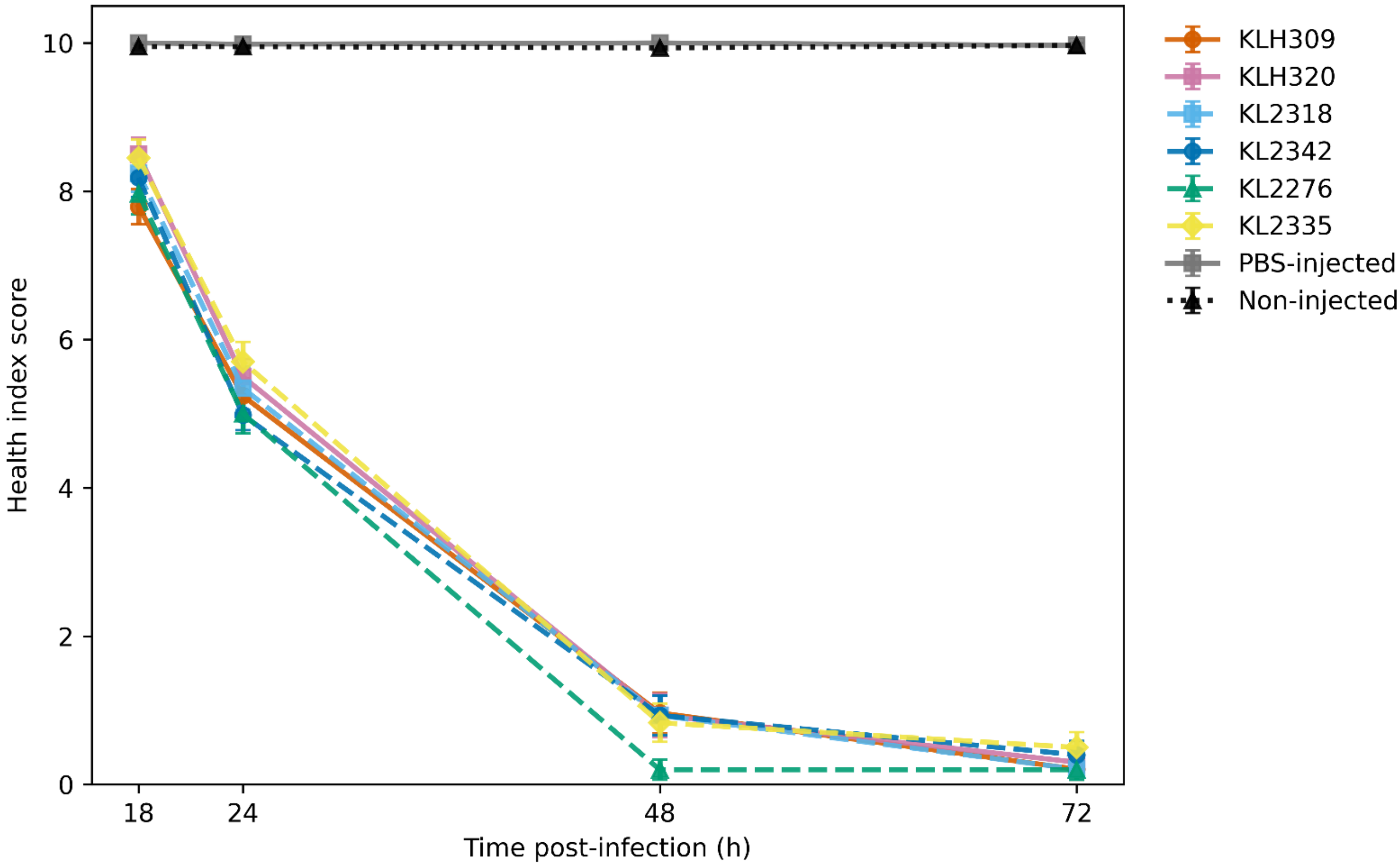

Supplementary Fig. S4. *Galleria mellonella* health index scores following infection with representative clinical and environmental isolates of *L. pneumophila*. Larvae were infected with approximately 10^6 CFU per larva and monitored at 18, 24, 48, and 72 h post-infection. Health index scores were evaluated using a previously described 10-point scoring system (42). Data represent means ± SEM from three independent experiments. No significant differences in health index scores were observed among isolates at any evaluated time point (Kruskal–Wallis test).
